# Microshunt Matchup: XEN vs. PRESERFLO in the Arena of Primary Open-Angle Glaucoma

**DOI:** 10.7759/cureus.95252

**Published:** 2025-10-23

**Authors:** Mohammad Ayoub, Ahmed Alnahrawy

**Affiliations:** 1 Geriatrics, Croydon Health Services NHS Trust, London, GBR; 2 Ophthalmology, Western Eye Hospital, Imperial College Healthcare NHS Trust, London, GBR

**Keywords:** intraocular pressure, minimally invasive glaucoma surgery, preserflo microshunt, primary open-angle glaucoma, xen gel stent

## Abstract

Minimally invasive bleb-forming procedures, such as the XEN® Gel Stent and the PRESERFLO™ MicroShunt, are surgical options used to lower intraocular pressure (IOP) in patients with primary open-angle glaucoma (POAG). This review compares the safety and efficacy of the two devices.

A literature search was conducted using Ovid Medline and Embase. Studies were included if they were published in journals with an impact factor above 1.5 and reported outcomes at least 6 months after implantation of XEN or PRESERFLO. Only adult patients with POAG were included. The main outcomes assessed were mean IOP reduction, change in the number of glaucoma medications, and the occurrence of key complications or further interventions.

Both devices demonstrated consistent and clinically meaningful IOP reduction in published studies. In a two-year, single-centre retrospective study, 41 eyes treated with XEN showed IOP reduction from 19.2 ± 4.4 mmHg to 13.8 ± 3.8 mmHg (a 28% reduction). In the same study, PRESERFLO in 41 eyes lowered IOP from 20.1 ± 5.0 mmHg to 12.1 ± 3.5 mmHg (a 39% reduction). The average number of medications decreased by approximately 62% with XEN and 69% with PRESERFLO. After 24 months, about 62% of XEN eyes and 64% of PRESERFLO eyes no longer required medications. Early hypotony, defined as IOP ≤ 5 mmHg, occurred in 24% of XEN and 39% of PRESERFLO cases. Persistent hypotony was reported in about 8% of XEN cases. Needling was more common after XEN (20%) than after PRESERFLO (5%). At two years, XEN studies showed around 28% IOP reduction, whereas PRESERFLO studies reported IOP lowering of about 35-39% over the same period.

In conclusion, both PRESERFLO and XEN are effective options for managing POAG. However, PRESERFLO may offer more consistent IOP reduction, greater medication-use reduction, and fewer post-operative interventions, making it a potentially more reliable long-term option.

## Introduction and background

Glaucoma is one of the leading causes of blindness worldwide, occurring due to damage to the optic nerve and cupping of the optic disc, which leads to progressive visual field loss. This damage is associated with the loss of retinal ganglion cells and elevated intraocular pressure (IOP) [1]. In 2010, glaucoma affected approximately 2.1 million people globally [2]. By 2040, the number of people with glaucoma is expected to rise to 111.8 million [1]. Primary open-angle glaucoma (POAG) is the most common subtype of glaucoma [3].

Glaucoma management has evolved significantly over the past four decades. The Tube Versus Trabeculectomy (TVT) study supported the increasing use of glaucoma drainage implants (GDIs), even as a first-line surgical treatment. Between 2008 and 2016, the total number of glaucoma surgeries in the United States increased by 14.7%, from 294,990 to 338,230. Over the same period, the number of trabeculectomies performed decreased by 11.7%, while minimally invasive procedures increased by 426% [4]. In the United Kingdom, glaucoma accounts for approximately 20% of all eye clinic visits, with over 1 million appointments each year, and these numbers are projected to rise [5].

The aim of this study is to assess and highlight the clinical outcomes, safety, and effectiveness of two bleb-forming minimally invasive procedures, the XEN® Gel Stent and the PRESERFLO™ MicroShunt.

Materials and methods

**Figure 1:**
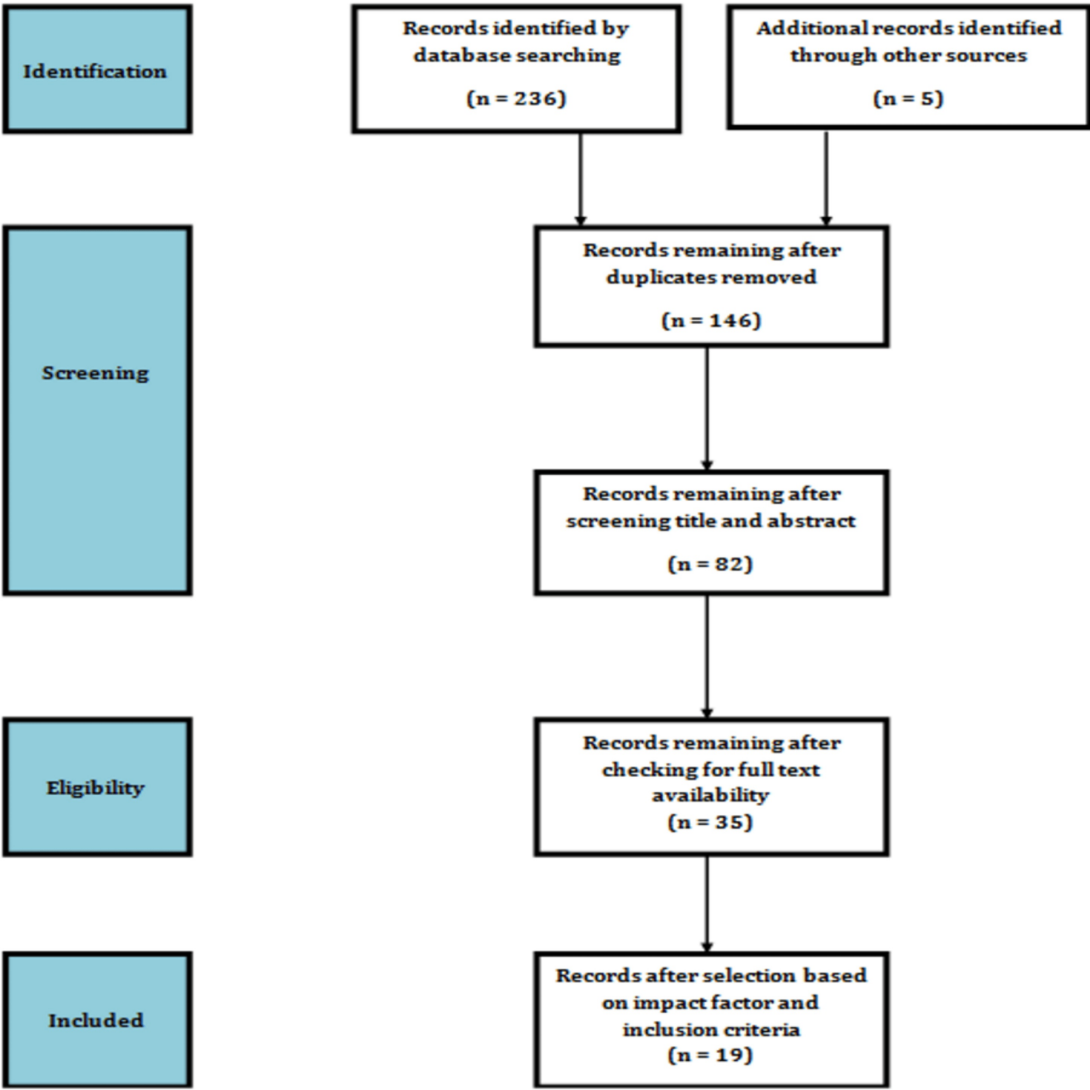
Diagram depicting the process for the selection of studies.

This review was conducted using Ovid Medline and Ovid Embase. Studies were included if they reported clinical outcomes following XEN Gel Stent or PRESERFLO MicroShunt implantation, were published in journals with an impact factor greater than 1.5, and had at least six months of follow-up. Only adult patients with POAG were included. Studies on other glaucoma subtypes or paediatric age groups were excluded. The primary outcomes assessed were IOP reduction, reduction in medication use, and major complications or subsequent surgical interventions. Keywords used in the literature search included: minimally invasive glaucoma surgery, XEN Gel Stent, PRESERFLO MicroShunt, POAG, and IOP. The study selection process is illustrated in Figure 1.

## Review

Results

PreserFlo MicroShunt

The PRESERFLO™ MicroShunt (Santen Pharmaceutical Co., Ltd.) is a soft and flexible stent made from a biocompatible material called SIBS (poly(styrene-block-isobutylene-block-styrene)). It is inserted into the eye using an ab-externo technique. Due to its flexibility, SIBS adapts well to the eye’s shape and reduces the risk of scleral erosion. It is also biocompatible and does not break down over time, which means it causes very little inflammation and does not lead to scarring or capsule formation [6]. The device works by allowing aqueous humour to drain from the anterior chamber of the eye into a space beneath the conjunctiva and Tenon’s capsule, forming a small fluid-filled area known as a bleb [7].

XEN Gel Stent

The XEN® Gel Stent (Allergan, an AbbVie company) is a soft, permanent implant inserted via an ab-interno approach. It allows aqueous fluid to drain from the anterior chamber into the subconjunctival space through a channel in the sclera. It is made from porcine-derived collagen cross-linked with glutaraldehyde. The stent is hydrophilic and is firm when dry but soft and flexible when hydrated, allowing it to conform well to surrounding tissues. It follows an S-shaped curve through the sclera and forms a natural bend of about 35 degrees beneath the conjunctiva and Tenon’s capsule. Its flexibility reduces pressure on the conjunctiva, lowering the risk of erosion. The stent is delivered using a preloaded 27-gauge needle and is deployed by sliding a control on the injector, placing the device in position [8].

XEN in direct comparison to PRESERFLO

XEN® Gel Stent Compared to PRESERFLO™ MicroShunt Implantation for POAG: Two-Year Results

This retrospective comparative case series assessed outcomes in 82 eyes (41 XEN and 41 PRESERFLO MicroShunt) over a 24-month follow-up period. The study population included 64 patients. The mean preoperative IOP was 19.2 ± 4.4 mmHg in the XEN group and 20.1 ± 5.0 mmHg in the MicroShunt group. At 24 months, IOP reduced to 13.8 ± 3.8 mmHg (a 28% decrease) in the XEN group and to 12.1 ± 3.5 mmHg (a 39% decrease) in the MicroShunt group. Medication use also decreased from 2.5 ± 1.4 to 0.9 ± 1.2 in the XEN group (62% medication-free) and from 2.3 ± 1.5 to 0.7 ± 1.1 in the MicroShunt group (64% medication-free). Early hypotony (IOP ≤ 5 mmHg) occurred more frequently in the MicroShunt group (39%) than in the XEN group (24%). However, late complications such as persistent hypotony (8%) and stent curling (15%) were observed only in the XEN group. Postoperative interventions, including bleb needling and Micropulse Transscleral Cyclophotocoagulation (MP-TSCPC), were more frequent in the XEN group. A summary of IOP and medication reduction data, along with adverse effects, is presented in Table 1 [9].

**Table 1:** XEN gel stent compared to PRESERFLO™ microShunt for POAG: two-year results. Source: Reference [9] Study type: Retrospective cohort study; Duration: 2 years; Number of centers: 1 (Netherlands); Total population: 82 eyes (41 XEN, 41 PRESERFLO). Trab: Trabeculectomy; IOP: Intraocular Pressure; MED: Medication; Popn: Population; No.: Number; AC: Anterior Chamber.

Group	Pre-op IOP (mmHg)	Post-op IOP (mmHg)	IOP Decrease	Pre-op MED	Post-op MED	MED Decrease	Early Adverse effects (% of the popn)	Xen	PreserFlo
XEN (n=41)	19.2 ± 4.4	13.8 ± 3.8	-5.4 (28.1%)	2.5 ± 1.4	0.9 ± 1.2	-1.6 (64%)	Hypotony (IOP ≤ 5 mmHg)	24	39
							Hypotony requiring AC reformation	5	2
							Early hyphaema	22	20
							Choroidal detachment	2	2
PreserFlo (n=41)	20.1 ± 5.0	12.1 ± 3.5	-8.0 (39.8%)	2.3 ± 1.5	0.7 ± 1.1	-1.6 (69.6%)	Late Adverse effects (% of the popn)	Xen	PreserFlo
							Hypotony	8	0
							Curling of XEN	15	0
							Migration of XEN	2	0
							Tube occlusion	0	2
							Ptosis	0	2

PRESERFLO MicroShunt

Comparison of PRESERFLO and Trabeculectomy in POAG

This was a two-year, multicentre, prospective, randomized study. The one-year outcomes of PRESERFLO and trabeculectomy in 527 patients with POAG were analyzed. Of these, 395 received PRESERFLO and 132 underwent trabeculectomy. At one year, mean intraocular pressure (IOP) dropped from 21.1 ± 4.9 mmHg to 14.3 ± 4.3 mmHg in the PRESERFLO group and from 21.1 ± 5.0 mmHg to 11.1 ± 4.3 mmHg in the trabeculectomy group. The mean number of medications reduced from 3.1 ± 1.0 to 0.6 ± 1.1 for PRESERFLO and from 3.0 ± 0.9 to 0.3 ± 0.9 for trabeculectomy. Adverse events included more cases of elevated IOP requiring treatment in the PRESERFLO group (32.9% vs. 13.7%). However, hypotony (6.1% vs. 13.7%) and visual field deterioration (10.4% vs. 15.3%) were lower in the PRESERFLO group. Loss of ≥2 lines of BCVA occurred less frequently in the PRESERFLO group (6.1% vs. 10.7%). A summary of data on IOP and medication reduction, along with adverse effects, is presented in Table 2 [10].

**Table 2:** Ab-externo MicroShunt versus trabeculectomy in primary open-angle glaucoma: one-year results. Source: Reference [10]. Study type: Prospective, multicentre, randomized controlled trial; Duration: 2 years (1-year results presented); Number of centers: 29 sites (24 in the USA, and 1 each in France, Italy, the Netherlands, Spain, and the UK); Total population: 527 patients (395 PRESERFLO, 132 Trabeculectomy). Trab: Trabeculectomy; IOP: Intraocular Pressure; MED: Medication; Popn: Population; No.: Number; VF: Visual Field; BCVA: Best-Corrected Visual Acuity.

Group	Pre-op IOP (mmHg)	Post-op IOP (mmHg)	IOP Decrease	Pre-op MED	Post-op MED	MED Decrease	Adverse Effects (% of Population)	PRESERFLO	Trab	p-value
PRESERFLO (n = 395)	21.1 ± 4.9	17.4 ± 3.7	-3.7 (17.5%)	3.1 ± 1.0	0.6 ± 1.1	-2.5 (80.6%)	Increased IOP (≥ 30 mmHg)	32.9	13.7	0.000033
							VF worsening	10.4	15.3	0.009565
Trab (n = 132)	21.1 ± 5.0	11.1 ± 4.3	-10.0 (47.4%)	3.0 ± 0.9	0.3 ± 0.9	-2.7 (90%)	Hypotony	6.1	13.7	0.109848
							Loss of BCVA > 2 lines	6.1	10.7	0.121698
							Hyphema	6.1	2.3	0.184687

Efficacy and Safety of PRESERFLO vs. Trabeculectomy

In this prospective cohort study, 60 eyes with POAG were treated with either PRESERFLO or trabeculectomy (30 eyes each). At one year, both groups achieved a significant IOP reduction without medication, PRESERFLO from 16.2 to 10.5 mmHg and trabeculectomy from 17.6 to 11.1 mmHg. Preoperative medication use dropped from 4 to 0 in both groups. No Seidel-positive leaks were reported in the PRESERFLO group, whereas six were seen in the trabeculectomy group. PRESERFLO had a higher incidence of hypotony (16 vs. 9 cases) and slightly more cases requiring anterior chamber reformation (8 vs. 7). Choroidal effusion occurred in eight eyes in both groups. PRESERFLO had no cases of hypotony maculopathy, hyphema, or laser suture lysis, while trabeculectomy had one, one, and thirteen cases, respectively. Bleb needling was required in one PRESERFLO case versus four in the trabeculectomy group. A summary of data on IOP and medication reduction, along with adverse effects, is presented in Table 3 [11].

**Table 3:** PRESERFLO™ MicroShunt versus trabeculectomy: one-year results on efficacy and safety. Source: Reference [11]. Study type: Prospective interventional cohort study; Duration: 1 year; Number of centers: 1 (Dresden, Germany); Total population: 60 eyes (30 PRESERFLO, 30 Trabeculectomy). Trab: Trabeculectomy; IOP: Intraocular Pressure; MED: Medication; Popn: Population; No.: Number; AC: Anterior Chamber.

Group	Pre-op IOP (mmHg)	Post-op IOP (mmHg)	IOP Decrease	Pre-op MED	Post-op MED	MED Decrease	Adverse Effects (No. of Population)	PRESERFLO	Trab	p-value
PRESERFLO (n = 30)	16.2	10.5	-5.7 (35.2%)	4	0	-4 (100%)	Seidel-positive leakage	0	6	0.0237
							Hypotony (< 5 mmHg)	16	9	0.1154
							Hypotony requiring AC reformation	8	7	1
Trab (n = 30)	17.6	11.1	-6.5 (36.9%)	4	0	-4 (100%)	Laser suture lysis	0	13	0.00005
							Hyphema	0	1	1
							Bleb needling	1	4	0.3533

Two-Year Multicentre Study on PRESERFLO in POAG

This prospective, multicentre study evaluated 81 POAG patients undergoing PRESERFLO implantation. At baseline, mean IOP was 21.7 ± 3.4 mmHg. At two years, IOP decreased to 14.1 ± 3.2 mmHg. By the second year, medication use dropped from 2.1 ± 1.3 to 0.5 ± 0.9, with 73.8% of patients being medication-free. Common adverse events included elevated IOP (24.7%), keratitis (4.0%), pain, diplopia, cataract formation, and corneal contact by the device (each 1.2%). A summary of data on IOP and medication reduction, along with adverse effects, is presented in Table 4 [12].

**Table 4:** Safety and effectiveness of PRESERFLO in primary open-angle glaucoma: results from a 2-year multicenter study. Source: Reference [12]. Study type: Prospective, single-arm, multicenter clinical trial; Duration: 2 years; Number of centers: 6 European sites (France: 3, Netherlands: 1, Spain: 1, Switzerland: 1); Total population: 81 eyes. IOP: Intraocular Pressure; MED: Medication; Popn: Population; No.: Number.

Group	Pre-op IOP (mmHg)	Post-op IOP (mmHg)	IOP Decrease	Pre-op MED	Post-op MED	MED Decrease	Adverse Effects (% of Population)
PRESERFLO (n = 81)	21.7 ± 3.4	14.1 ± 3.2	-7.6 (35%)	2.1 ± 1.3	0.5 ± 0.9	-1.6 (76.2%)	Increased IOP
							Keratitis
							Pain
							Diplopia
							Cataract
							Device touching cornea

One-Year Safety and Efficacy of PRESERFLO

This retrospective multicentre study evaluated 100 eyes over 12 months. Baseline IOP was 21.5 mmHg (range 19-28 mmHg), which dropped to 13 mmHg at six months and remained stable at twelve months. Medication use decreased from three to zero. Reported complications included anterior uveitis (2%), peripheral corneal oedema (1%), iris incarceration (1%), and macular oedema secondary to CRVO (1%). A summary of data on IOP and medication reduction, along with adverse effects, is presented in Table 5 [13].

**Table 5:** Short-term safety and efficacy of PRESERFLO™ MicroShunt in glaucoma patients. Source: Reference [13]. Study type: Multicentre, retrospective cohort study; Duration: 1 year; Number of centers: 4 tertiary referral glaucoma centers across Europe; Total population: 100 eyes from 91 patients. IOP: Intraocular Pressure; MED: Medication; Popn: Population; No.: Number.

Group	Pre-op IOP (mmHg)	Post-op IOP (mmHg)	IOP Decrease	Pre-op MED	Post-op MED	MED Decrease	Adverse Effects (% of Population)
PRESERFLO (n = 100 eyes)	21.5	13	-8.5 (39.5%)	3	0	-3 (100%)	Anterior uveitis
							Peripheral corneal edema
							Iris incarceration
							Macular edema

PRESERFLO after Failed Trabeculectomy in POAG

This retrospective study assessed 31 pseudophakic POAG patients who underwent PRESERFLO after a failed trabeculectomy. Preoperative IOP was 24.12 ± 3.14 mmHg, which dropped to 12.56 ± 2.64 mmHg at one year. Medication use reduced from 3.29 ± 0.64 to 0.46 ± 0.77. Transient hypotony occurred in 19.3% (6 eyes), and choroidal effusion in 9.6% (3 eyes). All adverse events resolved without intervention. A summary of data on IOP and medication reduction, along with adverse effects, is presented in Table 6 [14].

**Table 6:** Efficacy and safety of PRESERFLO™ MicroShunt after a failed trabeculectomy in eyes with primary open-angle glaucoma. Source: Reference [14]. Study type: Retrospective, interventional, multicentre study; Duration: 1 year; Number of centers: 2 sites; Total population: 31 patients. IOP: Intraocular Pressure; MED: Medication; Popn.: Population; No.: Number.

Group	Pre-op IOP (mmHg)	Post-op IOP (mmHg)	IOP Decrease	Pre-op MED	Post-op MED	MED Decrease	Adverse Effects (No. of Population)
PRESERFLO (n = 31)	24.12 ± 3.14	12.56 ± 2.64	-11.56 (47.9%)	3.29 ± 0.64	0.46 ± 0.77	-2.83 (86.0%)	Transient hypotony
							Choroidal effusion

XEN gel stent

Two-Year Results of a Multicentre Study of the Ab-Interno Gelatin Implant in Medically Uncontrolled POAG

This prospective, non-randomized, multicentre clinical study followed 218 eyes with POAG over two years. Of these, 120 underwent XEN implantation alone and 98 received combined Phaco + XEN. In the XEN group, mean IOP reduced from 21.7 mmHg to 15.4 mmHg (29% reduction), and medication use dropped from 2.7 to 1.2 (55.6% reduction). In the Phaco + XEN group, IOP dropped from 21.0 mmHg to 14.9 mmHg (29% reduction) and medications from 2.5 to 1.0 (60% reduction). Adverse events included secondary surgical intervention (6.4%), hyphema (4.6%), device fracture (2.8%), and hypotony (2.3%) in the XEN group, while the Phaco + XEN group experienced choroidal effusion (1.8%), conjunctival erosion (1.8%), implant blockage (1.4%), eye pain (1.4%), and iritis (0.9%). A summary of data on IOP and medication reduction, along with adverse effects, is presented in Table 7 [15].

**Table 7:** Two-year results of a multicenter study of the ab-interno gelatin implant in medically uncontrolled primary open-angle glaucoma. Source: Reference [15]. Study type: Prospective, non-randomized, multicentre clinical study; Duration: 2 years; Number of centers: 21 sites across 8 countries; Total population: 218 eyes (120 XEN alone, 98 Phaco + XEN). XEN: XEN Gel Stent; Phaco: Phacoemulsification; IOP: Intraocular Pressure; MED: Medication; Popn: Population.

Group	Pre-op IOP (mmHg)	Post-op IOP (mmHg)	IOP Decrease	Pre-op MED	Post-op MED	MED Decrease	Adverse effects for both group	
Xen (n=120)	21.7	15.4	-6.3 (29%)	2.7	1.2	-1.5 (55.6%)	Secondary Surgical Intervention	14 (6.4%)
							Hyphaema	10 (4.6%)
							Device fracture	6 (2.8%)
							IOP increase	6 (2.8%)
							Hypotony	5 (2.3%)
							Choroidal effusion	4 (1.8%)
Phaco+Xen (n=98)	21.0	14.9	-6.1 (29%)	2.5	1.0	-1.5 (60%)	Conjunctival erosion	4 (1.8%)
							Implant blockage by iris	3 (1.4%)
							Eye pain	3 (1.4%)
							Blepharitis	2 (0.9%)
							Iritis	2 (0.9%)

*XEN 45 Gel Stent Implantation in Open-Angle Glaucoma: Five-Year Results of a Prospective Study* 

This prospective, single-centre interventional study in Switzerland followed 170 eyes over five years. Among them, 42 underwent XEN alone and 128 received Phaco + XEN. In the XEN group, IOP decreased from 19.6 mmHg to 12.5 mmHg (36.2%), and medications reduced from 2.0 to 0.8 (60%). The Phaco + XEN group showed a similar IOP drop (19.8 mmHg to 12.6 mmHg, 36.4%) and medication reduction (2.0 to 0.8, 60%). Adverse effects included needling in 49% of cases, secondary glaucoma surgery in 19.4%, IOP spikes > 30 mmHg (10.6%), hyphema (8.8%), and early hypotony (2.9%). Rare complications (< 1.2%) included choroidal effusion, stent blockage, corneal oedema, retinal detachment, and endophthalmitis. A summary of data on IOP and medication reduction, along with adverse effects, is presented in Table 8 [16].

**Table 8:** XEN® 45 gel stent implantation in open-angle glaucoma: five-year results of a prospective study. Source: Reference [16]. Study type: Prospective, single-centre, interventional study; Duration: 5 years; Number of centers: 1 (Switzerland); Total population: 170 eyes. XEN: XEN Gel Stent; Phaco: Phacoemulsification; IOP: Intraocular Pressure; MED: Medication; Popn: Population.

Group	Pre-op IOP (mmHg)	Post-op IOP (mmHg)	IOP Decrease	Pre-op MED	Post-op MED	MED Decrease	Adverse effects (% of the popn)	
XEN (n=42)	19.6 ± 7.1	12.5 ± 3.1	-7.1 (36.2%)	2.0 ± 1.3	0.8 ± 1.1	-1.2 (60%)	Needling	49
							Secondary surgery	19.4
							IOP spikes ≥ 30 mm Hg	10.6
							Hyphema	8.8
XEN+Phaco (n=128)	19.8 ± 7.0	12.6 ± 3.1	-7.2 (36.4%)	2.0 ± 1.3	0.8 ± 1.1	-1.2 (60%)	Early hypotony	2.9
							Choroidal effusion; IOL dislocation; Stent blockage	<1.2%
							Persistent hypotony; Corneal edema; Retinal detachment; Endophthalmitis	<0.6%

Comparing the Efficacy of Trabeculectomy and XEN Gel Microstent Implantation for the Treatment of POAG

In this retrospective, single-centre comparative cohort study involving 200 eyes (100 XEN, 100 trabeculectomy), outcomes were measured over one year. XEN reduced IOP from 24.5 mmHg to 16.6 mmHg (32.2%) and medication use from 3.0 to 1.4 (53.3%). Trabeculectomy showed a greater IOP decrease from 24.8 mmHg to 14.8 mmHg (40.3%) and medication drop from 3.3 to 1.3 (60.6%). Hypotony occurred significantly more often with XEN (30%) than trabeculectomy (16%) (p = 0.029). Needling was required in 42% of XEN and 22% of trabeculectomy cases (p = 0.004). Post-surgical interventions were more frequent after trabeculectomy (33%) than XEN (16%) (p = 0.009). A summary of data on IOP and medication reduction, along with adverse effects, is presented in Table 9 [17].

**Table 9:** Comparing the efficacy of trabeculectomy and XEN® gel microstent implantation for the treatment of primary open-angle glaucoma. Source: Reference [17]. Study type: Retrospective, monocentric, comparative cohort study; Duration: 1 year; Number of centers: 1 site; Total population: 200 eyes (100 XEN, 100 Trabeculectomy). XEN: XEN Gel Stent; Phaco: Phacoemulsification; IOP: Intraocular Pressure; Trab: Trabeculectomy; MED: Medication; Popn: Population.

Group	Pre-op IOP (mmHg)	Post-op IOP (mmHg)	IOP Decrease	Pre-op MED	Post-op MED	MED Decrease	Adverse Effects (% of Population)	XEN	Trab	p-value
XEN (n = 100)	24.5 ± 6.7	16.6 ± 4.8	-7.9 (32.2%)	3.0 ± 1.1	1.4 ± 1.5	-1.6 (53.3%)	Hypotony	30	16	0.029
							Choroidal detachment	14	9	0.375
Trab (n = 100)	24.8 ± 7.8	14.8 ± 4.0	-10.0 (40.3%)	3.3 ± 1.2	1.3 ± 1.4	-2.0 (60.6%)	Needling required	42	22	0.004
							Post-surgical interventions (e.g., suture lysis, bleb revision)	16	33	0.009

Efficacy and Safety at Six Months of the XEN Implant for the Management of Open-Angle Glaucoma

This retrospective, non-interventional, monocentric open-label study followed 107 eyes over six months. The XEN group (n = 77) showed IOP reduction from 20.4 mmHg to 15.4 mmHg (24.5%) and medication reduction from 3.0 to 0.5 (83.3%). The Phaco + XEN group (n = 30) had IOP reduction from 19.9 mmHg to 17.1 mmHg (14.1%) and medication drop from 2.3 to 0.9 (60.9%). Adverse effects in the XEN group included hypertony (16.8%), angled stent placement (14%), hyphema (11.2%), flat anterior chamber (3.7%), and transient hypotony (2.8%). Additional issues in the Phaco + XEN group included choroidal effusion (2.8%), cataract (2.8%), and macular oedema (1.9%). A summary of data on IOP and medication reduction, along with adverse effects, is presented in Table 10 [18].

**Table 10:** Efficacy and safety at six months of the XEN implant for the management of open-angle glaucoma. Source: Reference [18]. Study type: Retrospective, non-interventional, monocentric, open-label study; Duration: 6 months; Number of centers: 1 (single centre); Total population: 107 eyes from 97 patients. XEN: XEN Gel Stent; Phaco: Phacoemulsification; IOP: Intraocular Pressure; MED: Medication; Popn: Population.

Group	Pre-op IOP (mmHg)	Post-op IOP (mmHg)	IOP Decrease	Pre-op MED	Post-op MED	MED Decrease	Adverse effects (% of the popn)	
XEN (n=77)	20.4 ± 6.4	15.4 ± 5.3	-5.0 (24.5%)	3.0 ± 0.9	0.5 ± 0.9	-2.5 (83.3%)	Hypertony > 30 mmHg	16.82%
							Improper location, angled drain	14.02%
							Postoperative hyphema	11.21%
							Flat anterior chamber	3.74%
XEN+Phaco (n=30)	19.9 ± 5.6	17.1 ± 4.7	-2.8 (14.1%)	2.3 ± 0.8	0.9 ± 1.2	-1.4 (60.9%)	Transient hypotony <6mmHg (<1 month)	2.80%
							Choroidal effusion	2.80%
							Cataract	2.80%
							Macular Oedema	1.87%
							Chemosis	1.87%

Three-Year Clinical Outcome of XEN 45 Gel Stent Implantation versus Trabeculectomy in Patients with Open-Angle Glaucoma

This retrospective cohort study from Austria followed 142 eyes (58 XEN, 84 trabeculectomy) over three years. XEN reduced IOP from 23.4 mmHg to 13.8 mmHg (41.0%) and medication usage from 98.3% to 43.2% (55.1% reduction). Trabeculectomy showed a greater IOP drop from 25.1 mmHg to 11.2 mmHg (55.4%) and medication reduction from 97.6% to 2.0% (95.6%). Both groups had similar rates of hypotony with choroidal effusion (~13%). Other XEN complications included hyphema (6.9%), stent dislocation, fracture, and endothelial dysfunction (each ~1.7%). Trabeculectomy complications included IOL capture, glaucoma attacks, and maculopathy, all below 2%. A summary of data on IOP and medication reduction, along with adverse effects, is presented in Table 11 [19].

**Table 11:** Three-year clinical outcome of XEN® 45 gel stent implantation versus trabeculectomy in patients with open-angle glaucoma. Source: Reference [19]. Study type: Retrospective cohort study; Duration: 3 years; Number of centers: 1 (Austria); Total population: 142 eyes (58 XEN, 84 Trabeculectomy). XEN: XEN Gel Stent; Phaco: Phacoemulsification; IOP: Intraocular Pressure; Trab: Trabeculectomy; MED: Medication; Popn: Population.

Group	Pre-op IOP (mmHg)	Post-op IOP (mmHg)	IOP Decrease	Pre-op MED	Post-op MED	MED Decrease	Adverse effects (% of the popn)	Xen	Trab	P-value
XEN (n=58)	23.4	13.8	-9.6 (41.0%)	98.3%	43.2%	-55.1%	Hypotony with choroidal effusion	13.8	13.0	0.882
							Hyphaema	6.9	8.3	1.000
							Corneal Erosion	0.0	2.4	0.513
							Corneal Endothelial Dysfunction	1.7	0.0	0.408
Trab (n=84)	25.1	11.2	-13.9 (55.4%)	97.6%	2.0%	-95.6%	XEN dislocation	1.7	0.0	0.408
							XEN fracture	1.7	0.0	0.408
							IOL-Capture	1.7	0.0	0.408
							Glaucoma attack	1.7	0.0	0.408

Discussion

IOP Reduction

Both the PRESERFLO™ MicroShunt and the XEN® Gel Stent are effective in reducing IOP in patients with POAG. However, studies suggest that PRESERFLO may provide a greater reduction in IOP. In Beckers HJ et al. (2022), IOP dropped from 21.7 ± 3.4 mmHg to 14.1 ± 3.2 mmHg at two years, a 35% reduction. Quaranta L et al. (2021) studied patients with prior failed trabeculectomy and found an even larger drop from 24.12 ± 3.14 mmHg to 12.56 ± 2.64 mmHg (47.9%). In Bhayani R et al. (2023), IOP reduced by 8.5 mmHg over one year. Similar trends were reported by Jamke M et al. (2023) and Baker ND et al. (2021), where IOP reductions ranged between 17.5% and 39.5%.

The XEN Gel Stent also lowers IOP, though to a lesser extent. For example, in Torbey et al. (2023), IOP decreased from 19.6 ± 7.1 mmHg to 12.5 ± 3.1 mmHg over five years. Buffault J et al. (2020) reported a decrease from 20.4 ± 6.4 mmHg to 15.4 ± 5.3 mmHg after six months, while Rauchegger T et al. (2024) showed a 9.6 mmHg (41%) reduction after three years. Although XEN is effective, the IOP reduction is generally smaller compared with PRESERFLO.

Reduction in Medication Use

The PRESERFLO MicroShunt showed strong results in reducing the number of glaucoma medications across multiple studies. In Beckers HJ et al. (2022), medication use dropped from 2.1 ± 1.3 to 0.5 ± 0.9 at two years, and 73.8% of patients discontinued all glaucoma drops. Jamke M et al. (2023) and Bhayani R et al. (2023) also reported that 100% of patients became medication-free at one year. Quaranta L et al. (2021) observed a decrease from 3.29 ± 0.64 to 0.46 ± 0.77, a reduction of 2.83 medications (86%).

The XEN Gel Stent also helped patients reduce their dependence on medications. Torbey J et al. (2023) reported a drop from 2.0 ± 1.3 to 0.8 ± 1.1 medications at five years, with 62.5% becoming medication-free. Buffault J et al. (2020) showed a decrease from 3.0 ± 0.9 to 0.5 ± 0.9 in six months. In the direct comparison by Scheres LM et al. (2021), both groups achieved similar reductions (approximately 1.6 medications), with 62-64% of patients medication-free after two years. These findings suggest that both devices significantly reduce medication use, but PRESERFLO demonstrates more consistent results across different patient populations.

Complications and Postoperative Interventions

Both devices are generally safe, but they exhibit distinct complication profiles. The PRESERFLO MicroShunt has been associated with a higher rate of early hypotony in some studies, for instance, Scheres LM et al. (2021) reported that 39% of patients (approximately 16 eyes) experienced hypotony (IOP ≤ 5 mmHg). However, other complications were relatively rare, and few patients required further surgical interventions. In the same study, only 5% required bleb needling. Other complications, such as uveitis and macular oedema, occurred in <2% of patients and typically resolved with conservative management. In contrast, the XEN Gel Stent demonstrated a higher frequency of device-related complications. In Scheres LM et al. (2021), 24% of patients developed early hypotony, 15% experienced stent curling, and 2% had stent migration (movement of the implant toward the anterior chamber). Needling was required in 20% of cases, and another 20% required MP-TSCPC, significantly more than in the PRESERFLO group (p = 0.029). Torbey J et al. (2023) also reported high rates of needling (up to 49%) and complications such as hyphema, IOP spikes, and flat anterior chambers. These findings indicate that XEN may require more frequent postoperative interventions and carries a higher risk of mechanical complications.

Direct Comparative Study

Scheres LM et al. (2021) directly compared the XEN Gel Stent and the PRESERFLO MicroShunt in a retrospective case series including 82 eyes (41 in each group) followed for 24 months. The study found that PRESERFLO achieved a greater IOP reduction, from 20.1 ± 5.0 mmHg to 12.1 ± 3.5 mmHg (a decrease of 8.0 mmHg), compared with XEN, which reduced IOP from 19.2 ± 4.4 mmHg to 13.8 ± 3.8 mmHg (a 5.4 mmHg decrease). These results support the notion that PRESERFLO may be more effective, particularly in patients with higher baseline IOP or more advanced disease.

Medication reduction was similar between the two groups, with approximately two-thirds of patients becoming medication-free at two years. However, PRESERFLO was associated with fewer device-related issues and required fewer postoperative interventions. For example, stent curling and migration occurred only in the XEN group.

## Conclusions

In conclusion, both the PRESERFLO™ MicroShunt and the XEN® Gel Stent are safe and effective options for managing POAG. However, evidence from the head-to-head study comparing the two devices suggests that PRESERFLO may provide better IOP control, more consistent medication reduction, and fewer long-term complications or postoperative interventions. Notably, 20% of patients who underwent XEN Gel Stent implantation required MP-TSCPC, compared with none in the PRESERFLO group. While the XEN Gel Stent remains a valuable minimally invasive alternative, the PRESERFLO MicroShunt appears to be the more reliable option for sustained, long-term glaucoma management.
